# Exclusion by design: The undocumented 1.5 generation in the U.S

**DOI:** 10.3389/fsoc.2023.1082177

**Published:** 2023-03-03

**Authors:** Linda E. Sanchez

**Affiliations:** Department of Anthropology, University of California, Irvine, Irvine, CA, United States

**Keywords:** DACA, 1.5 generation, undocumented immigration, liminal legality, undocumented youth, immigration policy

## Abstract

This article focuses on Mexican individuals who grew up in the U.S. (1.5 generation) without documents and were not able to benefit from Deferred Action for Childhood Arrivals (DACA) or who were unable to renew their DACA. A 2012 Executive Action by former president Obama, DACA gave some undocumented youth relief from deportation and a 2-year renewable work permit provided they met certain criteria. Undocumented individuals DACA failed to reach have generally been overlooked in immigration research in favor of examining how DACA recipients' lives have been transformed by DACA. This project helps fill this gap by examining life outside of DACA, and how the program acted as an internal U.S. border of exclusion for many. This research also aids in understanding the impacts of changing government policies on vulnerable populations, especially those who are in some respects made even more vulnerable by their faith in the government, fear of the government, or are actively excluded from government programs. This investigation is part of a study that compares 20 DACA beneficiaries to 20 individuals without DACA. Through ethnographic methodologies and one-on-one interviews, this article examines the 20 research participants who fall outside DACA. It investigates why people who qualified for DACA did not apply, barriers to applying/renewing, and how members of the 1.5 generation were excluded from the program by restrictions such as date of arrival requirements. The article discusses what it means for research participants to live outside of DACA, and how they see their lives because they do not have DACA while others do. For example, what does it mean to age out of qualifying for DACA? What actions did individuals then take regarding their lack of legal status?

I recently got my wisdom teeth taken out, and then they [the pharmacist] asked me for an ID for the pain medication prescription. I had to tell the pharmacist that I don't have an ID. You know, it sucks because I don't even have an ID. This is the same reason why I can't go anywhere with my friends since so many places are 21 and over. Oh, I don't even tell my friends about my situation. You know, I only tell my very close friends, but I don't tell everyone my situation. I can't be like, “oh, no, I can't go out with you because I don't have an ID, I don't even have a birth certificate.” The times that I have told some of my friends, they don't believe me. They think that I'm joking around, and I'm just like, dude, I really don't have any ID. You guys don't understand my situation because you guys are citizens here or have DACA. I don't have any of that. It just sucks.

-Julie (Interview #40)

## Introduction

Julie was born in Mexico, and her parents brought her to the United States when she was only 3 years old. Although Julie is now 22 years old and grew up in the U.S. (Orange County, California), she is undocumented. Individuals who, like Julie, were born in a different country but raised in the U.S. are known as the 1.5 generation (Portes and Rumbaut, 2001; Rumbaut, 2004). In 2012 the executive action of Deferred Action for Childhood Arrivals (DACA) gave some undocumented youth a 2-year renewable work permit (these individuals are often referred to as Dreamers), but many were excluded through requirements like age cut-offs. Julie tried to apply to the DACA program but was unable to because she does not have a birth certificate or any type of government identification (more on her story later). I argue that DACA's strict requirements and qualification criteria such as those faced by Julie act as an internal border excluding many in the 1.5 generation from incorporation and participation into U.S. society.

National borders are traditionally defined through physical spaces as in the edge or boundary separating one country from its geographic neighbor like in the U.S.-Canada border crossing. These spaces are often the sites of extreme violence as evidenced by the more than 2,600 bodies found since 2000 in the U.S.-Mexico border state of Arizona alone (De Leon, 2015). In the last few decades however, the U.S. has brought its national borders from the boundaries of its physical peripheries to the interior of the country by enforcing programs that impact everyday life like the Secure Communities initiative, local police agreements with the Department of Homeland Security (DHS), and setting up DUI checkpoints, immigration checkpoints, and home and work raids (Menjívar, 2014; Gonzales and Raphael, 2017). These have resulted in countless arrests, detentions, and deportations, leaving immigrant communities fearful, left out of essential resources needed for survival, and feeling like outsiders in their own neighborhoods (De Genova, 2002). As Mezzadra and Neilson (2012) demonstrate, the proliferation of internal borders “are no less violent or discriminating than more traditional forms of bordering” (2012, p. 70).

This article explores the reasons why certain undocumented Mexican individuals of the 1.5 generation living in Southern California chose not to apply or could not apply for DACA. Most research on the undocumented 1.5 generation centers on individuals who received DACA, and how their lives have been positively transformed by the program. Little is known about the individuals DACA failed to reach, barriers faced when applying to the program, and the negative consequences of DACA such as forced name alteration (Sanchez, 2018, Forthcoming). This article helps fill this gap by focusing an anthropological lens on the day-to-day lives of research participants and examining how immigration law, policy, and programs impact lived experiences. It adds to studies of scholars like Vilchis Díaz (2021) on Dreamer subjectivities and how DACA in some ways reinforced exclusion of undocumented migrants (Perez Huber, 2015; Aranda et al., 2020; Menjívar, 2023). Furthermore, I conceptualize internal borders not as unintended consequences of immigration law, but as carefully crafted by the nation state in order to exclude through things like arbitrary requirements embedded in policy.

This paper focuses on 20 Mexican individuals who were left out of the DACA program and their daily lives through an anthropological lens and ethnographic methodologies. The main concept outlining the theoretical framework of this article is everyday bordering as posited by Yuval-Davis et al. (2018) to encompass the shift in recent immigration enforcement from the outer territorial border to the interior of a country. Internal bordering is caried out through mechanisms such as restrictive legislation, internal immigration checkpoints, and even through the expectation that ordinary citizens have a duty to become informal border-guards by overseeing documents at schools and jobs, as well as reporting suspected undocumented immigrants.

The second concept outlining the theoretical framework of this article is legal violence, which is defined as the “instances in which laws and their implementation give rise to practices that harm individuals physically, economically, psychologically, or emotionally” (Menjívar and Abrego, 2012, p. 11). Legal violence occurs when laws that are supposed to protect rights simultaneously enable marginalization and ill treatment of certain groups. Legal violence often manifests itself as a kind of political violence that operates through neglect. Important to the concept of legal violence is abject status, a term utilized to describe the “casting away of individuals and populations” as if they were disposable objects, which “shapes (or perhaps delimits) their social, economic, and biological life” (Gonzales and Chavez, 2012, p. 256). The abject designates those who have been rendered “other” in society through intersectionalities of race, gender, nationality, legal status, and many other categories (Butler, 1999; Chavez, 2008; De Genova, 2008).

As evident by Julie's testimony at the beginning of this article, quotidian life can be a real struggle when one is undocumented. By excluding Julie and others like her, the requirements act as an internal border preventing Julie from full participation in the society she grew up in. Things that most people would consider mundane occurrences like picking up prescription medications or going out with friends to places that are 21-and-over are completely off limits for Julie. She describes her situation as a constant feeling of being stuck. Other research participants shared that they do not travel outside county limits for fear of immigration checkpoints or that they are forced to miss out on internships or better paying jobs despite having all required qualifications due to their lack of legal status.

This paper sheds light on the impacts of changing government policies on vulnerable populations, especially those who are in some respects made even more vulnerable by actively being excluded from government programs. DACA is a good example of changing immigration government policy, and its volatility stems in part from the fact that it is not a law but an executive action, which leaves it open to being rescinded. This became a reality when the Trump administration rescinded DACA in September 2017 (Romo et al., 2017), thereby unleashing several battles in district and federal courts and politicizing the plight of the undocumented 1.5 generation even more (American Immigration Council, 2021). As it stands now, the U.S. government is not accepting any first-time applications. Only those who already have DACA may renew their work permits. This prevents many who qualify from benefiting from the program. Such restrictions paired with the insecurity of the ever-changing nature of the program, strict program requirements, an expensive application fee, and fear of government keeps people who grew up in the U.S. on the outside of society.

Through ethnographic methodologies including in-depth interviews of day-to-day struggles due to lack of legal status, I demonstrate how the undocumented 1.5 generation is contained as bodies at the border since they are actively excluded from full U.S. societal incorporation, and are, as Coutin puts it, “physically present but legally absent” (Coutin, 2007, p. 9). To demonstrate this, I begin by providing the methods utilized in this article. This is followed by a section outlining all the requirements one must meet in order to qualify for DACA, and how these acted as an internal border leaving many in the undocumented 1.5 generation without protection. Next, I give a brief overview of DACA's history and recent legal battles, followed by the demographics of DACA beneficiaries. After, I analyze the group of individuals who qualified but did not apply, which includes Julie's story. The second group I focus on are those who grew up in the United States, but do not qualify for DACA. The third group is made up of those who at one point had DACA, but for various reasons were unable to renew DACA and now fall outside of the program's protection. The fourth and last group I examine are research participants who applied to DACA but were denied. I end by making final observations and offering closing thoughts in the “Discussion and Conclusion” section.

## Methods

The data utilized in this article is part of a doctoral dissertation study in anthropology at the University of California, Irvine (UCI) made up of 40 interviews that compares individuals with DACA to those without. Ethnographic fieldwork was conducted in San Diego County and Orange County from September 2017 to April 2021 through confidential one-on-one interviews and participant observation. The majority of interviews were conducted from December of 2019 to March of 2021. This area in Southern California is home to one of the largest populations of DACA eligible individuals in the country (~60,000 people) (Cantor, 2015).

The University of California Irvine's Human Subjects Institutional Review Board (IRB) has approved the research methods. In order to protect research participants and to ensure confidentiality, each participant was given a pseudonym. No identifiers were collected during the interviews, and each interviewee is given a code number (e.g., interview 1). In addition, signed consent has been waived in favor for verbal consent by the IRB to further protect research participants by keeping their identity anonymous. Interviews, with consent, were recorded on digital recorders, and here too, no identifiers are recorded, merely “interview 1,” etc.

Most research participants for this study were recruited from a DREAMER resource center where I volunteered (helping with things such as tutoring, creating flyers for services offered, helping put on events, and more). Research participants would tell their friends and family about my study thereby recruiting additional research participants through word-of-mouth. Research participants were interviewed utilizing semi-structured open-ended questions. Interviews, ranging in duration from 45 min to 2 $\frac{1}{2}$ h, were recorded on digital recorders and transcribed. Narrative data analysis included coding the transcribed interviews and searching for thematic categories using MAXQDA, a qualitative data analysis computer program. With the outbreak of the COVID-19 pandemic, the research moved to a virtual platform as well as phone interviews.

The age range for this study's participants was 18 to 52 years old, but most participants (28 individuals) were between the ages 18–26. They broke down by gender as following, 26 research participants were female and 14 were male. For the ones without DACA protection, 13 were female and 7 were male. The same was true for those with DACA protection, 13 participants were female and 7 were male. According to recipient statistics in the U.S., there are slightly more females with DACA, which is reflected in the participant demographics of this study. Figures by USA Facts (2020) demonstrate that 53 percent of DACA recipients are female and 47% are male. The current average age of Dreamers is 28 with a large amount of individuals (203,890) between the ages of 21–25, followed by the age group of 26–30 years old (191,580) (American Immigration Council, 2021).

### DACA restrictions as internal borders and DACA beneficiaries

Border enforcement manifests insidiously through strict requirements embedded in immigration programs. DACA consideration is only given to immigrants who meet the rigid age restrictions of having arrived in the U.S. before their 16^th^ birthday and who were under 31 years of age when the program was announced on June 15, 2012. Eligibility also requires that applicants must have continuously resided in the U.S. since June 15, 2007 up to the present time, and must have had no lawful status on June 15, 2012. Additionally, there is an education and/or military service requisite that demands applicants be currently enrolled in school (or have returned to school), graduated from high school, obtained certificate of completion (e.g., GED), or be an honorably discharged U.S. veteran (Coast Guard or Armed Forces). Finally, to be considered for DACA, one must have not been convicted of a felony offense, a significant misdemeanor offense, multiple misdemeanor offenses, or otherwise pose a threat to national security or public safety.

Yuval-Davis et al. argue that the ever-increasing restrictions on qualification requirements for immigration programs is just one of many ways that wealthy countries (like the U.S., Britain, and Canada) displace borders and border controls relocating these to the inside of the country in a process they call “de- and re-bordering” (2018). These controls are essentially being carried out by anything, anyone, and anywhere–government agencies, private companies, document overseers, individual citizens, educational institutions, as well as court decisions, and mounting application and renewal fees (Yuval-Davis et al., 2018). These displaced borders seep into the daily life of immigrants in what Yuval-Davis et al. term “everyday bordering” since immigrants are blocked or are restricted from access to essential resources necessary for carrying out day-to-day life.

The strict harshness of DACA's qualification requirements is evident when the program is compared to past immigration programs like the Immigration Reform and Control Act of 1986 (IRCA) that did not have maximum age restrictions banning individuals from applying to amnesty. Furthermore, the education and/or military service requisite for DACA has no legal precedent in U.S. immigration law (Strauss, 2019; Zong and Batalova, 2019), and unfairly demands of the undocumented 1.5 generation something never before expected of any other immigrant group in U.S. history. Although some academics argue that strict criteria appeases anti-immigrant groups generally (Ngai, 2004; Olivas, 2020; Horton, 2020), the education requirement is notably stringent since high school graduation rates for undocumented youth are statistically low. Among undocumented people between the ages of 18–24, 40 percent have less than a high school education compared to 8 percent for those born in the U.S. (Passel and Cohn, 2009).

The strict requirements that prevent people from qualifying to DACA are not limited to the program. In fact, immigration opportunities are often limited in these ways. Anti-immigration pundits see it as the classical “floodgates” problem—in order to prevent opening the “floodgates” to many applicants, immigration programs are riddled with deadlines, age limits, minimum education qualifications, and a flurry of other ever-increasing criteria (Menjívar, 2014). Immigration programs and policy in the U.S. are becoming more restrictive and reducing or closing off pathways to legal residency and citizenship. However, this is not unique to the U.S. since there is a worldwide trend toward limited immigration. For instance, many countries in the global north such as Canada and England have been shifting to programs that only offer the type of liminal legality that DACA gives in something Canadian scholar, Smith, terms “global regimes of closure” (Smith, 2022). This leaves immigrants in precarious conditions since beneficiaries are not truly moved out of undocumentedness. Instead, immigrants are given temporary work permits that must be conditionally renewed and do not provide “legal status” or a pathway to citizenship.

It is estimated that ~1.2 million individuals in the U.S. out of a total undocumented population of 11 million were eligible for the conditionally renewed work permits that DACA offers (Vinopal, 2019). However, only 611,270 out of the 1.2 million were enrolled in the program at the end of March 2022 (National Public Radio, 2022). While there has not been much research on those who qualified but did not apply, it is hypothesized that many did not attempt to become DACA beneficiaries due to the high costs associated with the application, renewal, and lawyer fees (Gonzales et al., 2014). These claims are supported by the data collected from this study. Despite DACA recipients being < 1 percent of the total U.S. population, they pay 4 billion in taxes in every year, which is approximately a tenth of what the entire U.S. immigrant population contributes (Vinopal, 2019). The majority of DACA eligible, or 93 percent, are working or in school, and altogether DACA beneficiaries earned more than 23.4 billion dollars in 2017 (Vinopal, 2019).

National studies demonstrate that 69 percent of DACA beneficiaries saw their wages increase in part due to acquiring new employment that better fit their education, training, and career goals (Wong et al., 2017). Furthermore, 56 percent moved to a job with better working conditions. These numbers are in line with the experiences of this study's participants who have DACA as the majority saw an increase in their earnings and improved working conditions. Although DACA meant that beneficiaries had access to better paying jobs by being able to work legally and were able to contribute financially to their families and households (Gonzales et al., 2014; Wong and Valdivia, 2014; Teranishi et al., 2015), recipients continued expressing fear for their loved ones being detained and deported (Teranishi et al., 2015; Abrego, 2018). Although DACA allowed them to feel a sense of protection, they still stressed about the wellbeing of their undocumented relatives. Beyond figures of how beneficial DACA is and continues to be to those who have it or how much they contribute to the U.S. economy, this study examines the ways the 1.5 generation continue to be excluded.

### DACA's limitations, brief history, and recent legal developments

Despite DACA opening access to things such as social security cards, legal employment, and higher education, the program continues to impose limitations both formally and informally. Formally, DACA recipients are not able to travel in and out of the country. DACA individuals can acquire advance parole, a permit allowing travel outside the county under certain circumstances, but it is expensive, difficult to obtain, and does not guarantee being accepted back into country. Additionally, DACA recipients are barred from various jobs, especially government jobs, which are reserved for U.S. citizens and permanent legal residents. Informally, individuals with DACA are turned down from employment they legally qualify for. For example, a research participant in this study, Sara, obtained a job with T-Mobile, but they laid her off as soon as they learned she had DACA. The management at T-Mobile claims they did not want to risk hiring Sara because there is no guarantee that DACA will continue.

The reason the DACA program is at risk of not continuing is because it is an executive action and not a law. Former President Barack Obama instituted the DACA program through executive action in June of 2012 after the failure of the U.S. government to pass legal reform that would help undocumented individuals who were brought to the U.S. at a young age (Abrego, 2018). The DREAM Act was the legal reform that would have granted a pathway to citizenship for those in the undocumented 1.5 generation. The U.S. House of Representatives passed the DREAM Act in December of 2010, but the bill failed to pass in the senate (Olivas, 2020). DACA's intention was to provide those who would have qualified for the DREAM Act with some form of immigration relief. Because the program is not a law, those with DACA gained an in-between status, not fully legal since they do not have a pathway to citizenship, but not fully undocumented either, given that DACA provides some protections from deportation.

As mentioned in the introduction, the Trump's administration rescindment of DACA in June of 2017 ushered in a tumultuous period for DACA as legal battles at both the district and federal level were started. At times the courts have sided with the DACA program and at times have sided against the program. For instance, in June of 2020, the U.S. Supreme Court ruled that the Trump administration's rescindment of DACA was unlawful. However, more recently, in July of 2021, a U.S. District Court in Texas ruled the program as illegal on the grounds that it violates the Administrative Procedure Act (APA), a law regulating how federal agencies develop and issue policies (Department of Homeland Security, 2021). At the time of this writing, the latest court ruling on DACA came on October 5th, 2022, by the Fifth U.S. Circuit Court of Appeals issuing a decision stating that DACA could remain in place for now, but that no new applications would be accepted leaving hundreds of thousands who qualify without the opportunity to gain protection from the program (National Public Radio, 2022).

There is a possibility that DACA will once again go to the Supreme Court, and this has many of its supporters worried since the current U.S. Supreme Court is conservative leaning and unlikely to uphold DACA. NPR reports that data on Supreme Court rulings proves that the present Court is the most conservative in 90 years (Totenberg, 2022). For instance, the judges came to more conservative decisions in the 2022 terms than ever seen since 1931 (Totenberg, 2022). Although the future of DACA is uncertain in the current U.S. political climate, the Biden presidential administration released a memorandum reaffirming the federal government's continued support of the program stating that “the Secretary of Homeland Security, in consultation with the Attorney General, shall take all actions he deems appropriate, consistent with applicable law, to preserve and fortify DACA” (American Immigration Council, 2021).

### Qualified but did not apply

Julie, the research participant in the introduction, is one of 7 research participants in this study who qualified for DACA but could not apply. Like many other research participants who qualified but were unable to submit an application, she was missing one important required document. In her case, she was missing a birth certificate, but other participants found it difficult to prove through paperwork things like continual residence since 2007. Other research participants who qualified but did not apply said they lacked the financial resources. Individuals in this group often had more than one reason for not being able to apply. For example, they might not have had the money for their initial application and were missing required documents. It costs 495 dollars to apply to DACA plus about another 500 dollars for lawyer fees. The renewal fee one must pay every 2 years is also 495 dollars.

Julie's case is testament to the multitude of barriers individuals face trying to get DACA. In her interview, she states that her family did not have enough money for the initial application, and she does not have any form of government of identification because she does not have a birth certificate. Since Julie was born in a remote area of Mexico, and her family was not able to travel to the nearest city, a birth certificate was never issued for her. Additionally, Julie did not graduate high school because she became a teen mom at 16 years of age and was forced to drop out. However, Julie is currently enrolled in a GED program and hopes to be able to apply to DACA someday if the program opens to first-time applicants and she can obtain a birth certificate. She describes the predicament of her everyday life below:

I had just dropped off my son at school, I was driving, and accidentally made a U-turn where I wasn't supposed to. A cop stopped me and asked me for an ID. I told him I did not have an ID on me. I said I forgot it at home because I was scared. So, I went to court recently […] and the lady there said I have four months to bring my driver's license. If I prove that I have a license, they'll deduct the fine, and just charge me 25 dollars. So, I have until January, but if I don't bring my license, they're going to charge me more than 2,000 dollars. So, I'm just stuck. I'm just stuck in this situation.

-Julie (Interview #40)

Legal exclusion materializes in Julie's life by way of not having access to being able to drive legally, work legally, establish credit, have access to higher education, and much more. DACA's restrictive qualification criteria acts as an internal U.S. border forcing Julie to remain outside of legal incorporation. Because Julie cannot lawfully drive, she now faces the legal repercussions in the form of a 2,000 dollar fine. In her interview Julie states that she does not have the money to pay this fine. Her lack of finances is also connected to her legal exclusion since Julie remains working in the informal sector because she does not have a work permit, and earns less than minimum wage at the same small local restaurant she worked at throughout high school.

DACA's restrictive qualification criteria acts as an internal legal U.S. border that also translates socially in Julie's personal life by forcing her to remain outside of societal incorporation. She must also face the social ramifications that come from being legally excluded. Although Julie is physically present in the U.S., in many ways she is stuck outside of society. Growing up undocumented without any type of government identification was especially hard for Julie socially. Although Julie describes herself as culturally American, immigration program requirements act like borders preventing her from being part of many of the social rights-of-passages that American teenagers take part in such as getting a driver's license. Additionally, she was not able to move out with friends because she lacks the finances and a credit score. Instead, Julie lives in a crowded apartment with her son and other family members. This is why Julie describes her situation as “being stuck.” Barriers to inclusion are often invisible to those not living through situations like her own.

Other research participants who qualified for DACA but did not apply said they were unable to apply because they were afraid to give their information to the government because their family units include members with papers but also family members who are undocumented and cannot adjust their status. Nationwide, it is estimated that at least 16.7 million people are part of a mixed-status family (Mathema, 2017). Here “mixed-status” refers to a family unit consisting of at least one undocumented member and at least one other person with any immigrant legal status (i.e., legal permanent resident, U.S. Citizen) or temporary status (e.g., DACA).

Two research participants who did not apply to DACA over concerns for their undocumented family members was Stephanie (25 years old) and Yaneth (28 years old). They are two sisters from San Diego who at the time that DACA was announced still had valid visitor's visas. Their family had planned to overstay the visas and the two sisters feared for their parents' safety. Stephanie and Yaneth live with their parents in the same household. So, the two sisters wondered what would become of their parents when they gave up all of their information to the United States Citizen and Immigration Services (USCIS) in order to apply to DACA. Stephanie and Yaneth's parents were also fearful of what might happen if their daughters applied to DACA. Stephanie explains the fear her family felt below:

I knew that my parents were afraid, and this fear was transferred to me and my sister Yaneth. It was the fear of not knowing if Obama was going be re-elected back then in 2012. You know, even if he got re-elected. DACA is not a permanent fix, it is not even a law, it is an executive action.

-Stephanie (Interview #39).

As evident by Stephanie's statement, the uncertainty of liminal legality and precarity of conditional programs like DACA made many individuals who qualified weary to apply. In this way, the unpredictability of immigration programs can act to exclude those who need them and their families by default. The work of Heide Castañeda demonstrates that “the construction of illegality for some members in a family influences opportunities and resources for all” (2019, p. 16). Studies demonstrate that when at least one member of the family household can gain even temporary statuses like DACA, all the members benefit (Castañeda, 2019; Aranda et al., 2020). Individuals leveraged new opportunities established through DACA to help their families by, for example, obtaining a loan to purchase a car, driving family members, opening a bank account, picking up prescription medications, and much more. In this way, the gains are distributed in mixed-status families. While there is no doubt that this places extra responsibilities and thrusts new roles on DACA beneficiaries, it also makes a positive impact on their families (Castañeda, 2019; Aranda et al., 2020).

The fear that Stephanie feels toward government programs permeates other social aspects of her life. In this way, the legal exclusion she experiences materializes in her social life as well. Stephanie says that she does not share her legal status with anybody, not even close friends. She fears for her own wellbeing and that of her family's. Stephanie disclosed that although she was close to her professors at the university she attended, she did not share her status with them. Stephanie wanted to share her status and felt dishonest by not doing it, but ultimately made the decision to protect herself and her family. Stephanie's story demonstrates how the uncertainty of immigration programs that are temporary and conditionally renewed aid in maintaining internal borders. They operate as a technology of control since internal borders deter those who qualify from applying. Additionally, being excluded can then negatively affect important social relationships as it did for Stephanie with her professors and friends she could not go out with.

### Grew up in the United States but do not qualify for DACA

Besides those who qualified for DACA and could not apply, an equally large group of participants (7 individuals) in this study were people who were left out of DACA because they did not qualify. Despite having been raised in the U.S., the majority of these study participants were unable to meet the age requirements. Most research participants in this study were brought to the U.S. as infants or young children (under the age of 5 years old). This makes the age restrictions embedded in the program seem even more irrelevant. Many of those who were too old to apply to DACA were brought to the country as babies.

Antonio (41 years old) and Marissa (40 years old) are two study participants who did not qualify for DACA because they were slightly over the age limit of 31 when DACA was announced in 2012. They are husband and wife who grew up in Orange County and were very excited when DACA was announced because they both aspire to obtain better paying jobs to support their two young sons. A work permit would allow them to work legally and search for work outside their current line of work, the restaurant industry. Unfortunately, the age requirements acted as an internal border of exclusion preventing this from happening. Antonio expresses his frustration below:

We had all their requirements for DACA. We have everything because we graduated from a high school here in the U.S. Thank God. We have never been deported, we have never been to jail, nothing. So, we had everything but for the fact that we were just barely too old. When DACA was announced I was 33 and Marisa was 32. So, we couldn't apply, and I know a lot of other people that couldn't apply because of the age thing also. At first my mind was all like, “Finally, there is something that is going to help us.” So, we were excited […] and then when we didn't qualify for DACA, we were sad.

-Antonio (Interview #27)

Because Marissa and Antonio were not able to receive DACA, they remain in jobs that are precarious and do not pay well. One of their life goals is to purchase a new car and someday a home as well, but without the benefits that DACA grants, they shared that this is unrealistic. As mentioned in an earlier section regarding DACA beneficiaries, national studies show that individuals who gained a work permit through the program experienced a considerable raise in their earnings. This allowed 65 percent of national study respondents to purchase their first car, and 24 percent of respondents 25 years and older to become first-time homeowners (Wong et al., 2017). Unfortunately, one of the ways that legal exclusion is experienced by Marissa and Antonio is by not being able to make these larger purchases.

Not all research participants in this study who are part of the 1.5 generation but did not qualify for DACA missed out due to their age. One individual, Jose, who is 18 years old, arrived in the U.S. in 2010, 3 years after the date of arrival cut-off of June 15, 2007. Jose explains that not being able to have access to DACA has negatively impacted his schooling. He recently graduated high school, is attending community college, and hopes to someday transfer to a 4-year university as a math major. However, his access to financial aid and scholarships are limited because of his immigration status:

I actually think that because I do not have DACA, I missed out on big things. One of those big things is being able to work and bring in a steady income, especially me as a student. Books, tuition for classes, and materials all add up. For example, right now with the pandemic every student needs a computer because we're in online learning. I didn't have a computer. I can't work, so I can't buy a computer. I had to miss out on class. Sometimes I ask myself if I am going to have to drop out of college.

-Jose (Interview #30)

Although Jose has been physically living in the U.S. for over two decades, he is not allowed to participate legally in society. In this way, the border is extended far beyond the physical demarcation of the nation affecting his everyday life by creating vulnerability. In his interview, Jose describes his lack of access to higher education as one of the most difficult things about being undocumented. Attending community college is a major component of his daily life and internal borders seep into this personal space producing precarity. This is once again testament to the fact that legal exclusion translates into social exclusion. Jose states that growing up he always felt that school was a place where he could thrive and feel safe. However, now in college, he feels that school has transformed into a place where he often feels vulnerable and inadequate. It is not uncommon for migrants to enjoy legal inclusion in primary and secondary school, but depending on the state, undocumented students can be banned from attending college altogether (Bravo-Moreno, 2009; Gonzales and Chavez, 2012).

Antonio's, Marissa's, and Jose's experiences reflect how arbitrary age and date requirements act as internal borders excluding members of the 1.5 generation from stepping out of illegality. Furthermore, it demonstrates how dated the DACA program is and the need for it to be updated or for a new more inclusive program altogether. In 2012 when DACA was enacted as an executive action, the requirement of residing in the U.S. since 2007 seemed more sensible since 2007 was only a few years in the past. At the time of this writing the year 2007 will soon be two decades in the past. The fact that no law has yet been passed to protect individuals like Antonio, Marissa and Jose says a lot about the current political climate in the U.S. and attitudes toward immigrants. This also demonstrates how everyday bordering is often simply formulated trough inaction in order to exclude, and it is testimony to the violence this unleashes on individuals.

### Unable to renew DACA

Another way that immigration policy acts as an internal border for research participants in this study is through the liminality embedded in the DACA program. As mentioned previously, DACA must be conditionally renewed every 2 years and it is extremely expensive. Three individuals in this study do not have DACA protection because they were unable to renew their DACA. Participants cited the high cost of renewing and fear during the Trump administration as reasons for not renewing. Although only 3 individuals in this study were unable to renew, expired DACAs are a much larger issue. I met many individuals through immigrant organizations and at the Dreamer Center who fell out of DACA protection because they found renewal fees too expensive, or they were afraid of giving more personal information to the government especially during Trump's presidency.

The financial burden of expensive renewal fees prevented DACA recipients in this study and in the country in general from renewing their DACA, and now find themselves without protection once more. To make matters worse, USCIS under the former Trump administration proposed an increase for renewal fees from 495 to 765 dollars (Vinopal, 2019; Garcia, 2020). If this proposed 55 percent hike does take effect, it would be catastrophical for individuals trying to remain in the program. This fee increase would be especially difficult for families who have multiple individuals who are DACA beneficiaries as the renewal fees are per individual not per family. The Immigrant Legal Resource Center, an organization seeking to improve immigration policy and advance immigrant rights, released a statement expressing that a fee hike could make it even more complicated for DACA recipients to remain in the U.S. (Vinopal, 2019).

As previously noted, one of the most precarious things about the liminality of DACA is that it is not a law, and this caused constant anxiety in the life of study participants. The experience of 26-year-old research participant, Elizabeth, demonstrates how DACA's liminality (both in the sense of it's precarity and high financial cost) materializes as an internal border further excluding those without DACA protections. Elizabeth originally had DACA but did not renew it because she did not have enough money for the renewal. She shares that she thinks that she might have been able to borrow the money from friends and family. However, she was also fearful after Trump was elected president. Elizabeth explains the fear she felt during the Trump presidency and the anxiety over the uncertainty of the program ending:

I did not renew because there was a lot of people telling us not to renew because Trump got elected and he rescinded DACA. A lot of my friends were so paranoid, and I started listening to them. I was really scared and there were interviews going on the news. There were a lot of reports of undocumented people with DACA being deported. These reports were saying that some people were thinking that it wasn't okay, that we shouldn't renew. People thought that it was not a good idea to renew DACA because then they [the government] would track us down, and we would become easy targets for Trump's administration. So, I did not want them [border patrol] to come to my home and find where I'm at.

-Elizabeth (Interview #2)

For Elizabeth and other recipients, the benefits of DACA do not outweigh the underlying uncertainty of the program (Patler et al., 2021). Individuals living in legal limbo are constantly forced to interact with state agencies to renew their work permits. They must submit to fingerprint and retinal scans for FBI background checks to prove clean criminal records and are thus over-surveilled (Asad, 2023; Menjívar, 2023). These encounters make the borders of the nation tangible to the 1.5 generation because it reaffirms that they are conditionally in the country, only temporarily protected, and always being watched. Because she did not renew her DACA, Elizabeth now further experiences internal borders through various types of exclusion in her everyday life. One of the ways this manifests is *via* her limited access to higher education since she is barred from many types of financial aid. Elizabeth states that one of her main priorities is earning a bachelor's degree. However, she is ineligible for grants, fellowships, paid internships, and most scholarships. This greatly delimits her chances to earn enough community college credits to be able to transfer.

Ana, 22 years old from San Diego also did not renew her DACA. She states the lack of money as her reason for not renewing. Her parents brought her to the U.S. when she was 3 years old and before her work permit expired, she worked at Legoland and Sesame Place. Her jobs are two tourist attractions in Southern California and she really enjoyed working there, but is no longer able to since her work permit has expired. She discusses why she did not renew her work permit below and how much she misses working at her jobs:

I feel that during that time I realized that my dad was the only one paying for rent, bills, groceries and other expenses for our family. So, I felt like I didn't want to put another weight on him. I did not want to burden him further. I didn't want him to have to spend more money when we were already very low on money. So, I decided like, oh, you know what, I'll do it eventually, just not now. Now my work permit has expired, and I can no longer work at Legoland and Sesame Place. It really sucks. I wish I would have had enough money to renew it.

-Ana (Interview #13)

Both Elizabeth and Ana state money as a barrier to being able to renew their DACA. The high cost of immigration policy keeps many immigrants from moving out of undocumentedness or it forces them to return into the shadows by not being able to renew their work permits. These high costs of applying, renewing, and fee increases act as internal national borders to transitioning out and remaining out of illegality. Elizabeth and Ana are today more fearful than ever before because in addition to being undocumented, they must also worry about the fact that the government now has all their information—where they live, where they go to school, and who their previous employers were. Before DACA, they expressed that they had some sense of security in knowing that the government did not truly realize they existed. They feel that their expired DACA work permits puts a huge bull's eye on them and on the undocumented family members who live with them.

### Denied DACA

Beyond not being able to renew DACA, two research participants in this study are not currently protected from deportation because they applied to DACA but were denied. DACA only had less than a 1 percent denial rate, but both participants were denied DACA because of minor run-ins with the law when they were younger. In order to qualify for DACA, one must go through an intense background check to verify an immaculate record. Under the qualification criteria, USCIS states that an applicant must not have any significant misdemeanors, but what counts as a “significant” misdemeanor is not defined. In this section, I tell the story of Carlos who was denied DACA due to a minor run-in with the police when he was a minor in high school.

Carlos' family brought him to the U.S. without documents when he was only a 1-month-old baby. Carlos, now 21 years old, lives in Orange County, and works with his father installing drywall. He is a community college student and one of his personal goals is to help his dad purchase a house someday. Carlos states that his academic objective is to transfer to a 4 year university, and earn a master's degree in a field of STEM (science, technology, engineering, and math). However, a run in with the law when he was 14 years old is making Carlos' aspirations difficult to achieve. In November of 2013 Carlos found the key to a local community outdoor recreation facility. Carlos thought it would be fun to go back to the recreation facility after hours and shoot hoops at the basketball court. He recounts what happened that evening (some details have been changed to protect Carlos' identity):

For some reason, I decided to video record [on my phone] when I got to the place. But I wasn't trying to do anything bad, I just like found the key to it [the recreation facility]! So, I was just trying to get into the basketball court. That's all I was doing. And while I was doing that, I guess someone saw me and they called the police on me. And as a kid, you know, like, what do I do? I ran away, and they [the police] found me, and it was the gang unit that found me, and so they stopped me thinking I was part of a gang. Then the cops detained me, took me into the station, and they processed me. They [the police] charged me with attempted burglary. I think it is considered a misdemeanor.

-Carlos (Interview #35)

Carlos applied to DACA in 2015 and a few months later he received a letter from the DHS stating that he needed to explain what had transpired in November of 2013. He also needed to provide proof of whether he was sentenced, and if so, he needed proof of completing his sentence. However, Carlos was never sent to court. Instead, Carlos was required to take classes for delinquent juveniles. Upon completion, he received a certificate, but Carlos and his family moved residences a lot and this document along with his police report were lost. Therefore, Carlos had to formally request his police report, but he encountered unsurmountable bureaucracy and was extremely intimidated. His petition to obtain the police report was denied three times. Carlos explains what happened next:

At that point all I could do was send USCIS a letter explaining what had happened [in November of 2013]. After that, a few months later, USCIS sent me another letter saying that my application was denied. Honestly, I was really sad when I first found out that my DACA was rejected. I realized, I guess, how big consequences can be, like, how much the things you do… how much of a consequence they are when you are older. That was my first real realization that I shouldn't be doing stupid things. Uhm… honestly, I thought that since they [USCIS] denied my application that they were going to send out a deportation order right away. Well, I thought that was going to happen and I was really scared.

- Carlos (Interview #35)

Carlos is unsure if he can reapply to DACA because the letter he received from USCIS stating that his DACA was denied does not have much information, and he is too scared to ask questions. The last thing Carlos wants is to bring attention to himself because he still fears that USCIS will send out a deportation order. Like many research participants in this study, he is also afraid for undocumented family members in his household. In this manner, the U.S.-Mexico border is re-bordered internally for Carlos and his family through court decisions and police inaction to issue necessary paperwork. His story is testament to how administrative inaction can be used to passively exclude individuals from incorporation. Gilmore refers to this inaction as “organized abandonment,” through which the state controls and deprives some groups of social benefits (Gilmore, 2022). Similarly, Menjívar argues that the state creates social exclusion when it neglects devalued groups through disregard of bureaucratic responsibilities (2023).

Had Carlos been a U.S. citizen, the act of using the key he found to access the recreation facility would most likely not have much impact on his future chance for success. Perhaps his act of “trespassing” would have been regarded by the court simply as something that a young teenager did without really thinking about consequences. Unfortunately, that evening in November of 2013 is still haunting Carlos since his DACA was denied and he is consequently not able to work legally in the U.S. In this manner the police who charged him with attempted burglary, the staff who rejected his requests to acquire his criminal record, and the immigration officials who ultimately made the decision to deny Carlo's DACA application all become informal border guards impeding Carlos from moving out of undocumentedness.

Being forced to remain in illegality by informal border guards has repercussions far beyond Carlos' ability to work. His lack of legal status also makes him ineligible for paid internships (despite having various STEM certifications) and most school scholarships, financial aid, grants, and fellowships. Additionally, since Carlos is forced to work under the table, he cannot provide proof of income, which means that he cannot apply for a house loan. This breaks his heart because, as mentioned earlier, he really wants to help his father purchase a house. Carlos' father also works under the table, and neither can provide solid proof of being employed to a bank. In his interview, Carlos shared that the most disheartening thing about not being able receive DACA is not being able to work legally since it makes it almost impossible to become a homeowner.

## Discussion and conclusion

As DACA's future and that of individuals like Carlos hangs in the uncertain balance of future court decisions, it is important to remember that DACA's termination would mean that as a society, the U.S. would be shutting out members who are part of our communities. This would also be accompanied by a financial cost to the local and national economy as well as a blow to the U.S. labor force. Analysis by FWD.us estimates that if DACA is terminated and beneficiaries are allowed to keep their work permits until they expire, it would cost the U.S. 22,000 jobs a month, every month for the next 2 years (Connor, 2022). Put another way, this means 1,000 individuals would be forced to leave their jobs every business day for the next 2 years, which would be detrimental to communities and families (Connor, 2022). The end of DACA would also mean that every day for 2 years, nearly 1,000 immediate U.S. citizen family members will witness a loved one be put at immediate deportation risk, and their ability to stay in the U.S. would be greatly compromised.

The end of DACA would also mean continuing to leave out individuals from our society who know no other home than the U.S. In this way restrictive policy and court decisions would continue to act as internal borders of exclusion by design for the 1.5 generation. Excluding undocumented individuals from immigration policy ultimately leaves large populations in the shadows and outside the limits of societal inclusion. As evident by this study and mounting scholarly evidence, exclusion hampers immigrants educational prospects, employment opportunities, marginalizes them, and makes them live in fear for themselves and their families (Massey, 2008; Yoshikawa, 2011; Menjívar and Kanstroom, 2014; Menjívar, 2023). Additionally, restrictive immigration policy and court decisions artificially stifles and blocks legal immigration.

The 1.5 generation is one of many immigrant groups who are pushed further into precarity as the nation state utilizes any crisis event like September 11, 2001, a pandemic, or recession to bolster the “homeland security state” and strengthen controls in immigrant communities (Gonzales, 2013). These practices are parallel to what many political geographers are referring to as “internal bordering” (Dear, 2013). De Genova puts it best when he states, that in innumerable places of Mexican immigrants' day-to-day life “‘illegality' reproduces the practical repercussions of the physical border between the U.S. and Mexico” (De Genova, 2004, p. 161).

Regarding the current growing worldwide trend of immigration regimes that offer no pathways to citizenship, Hiroshi Motomura observes that immigrants are no longer intended to become future naturalized citizens. Instead, the rationale that has become much too common in countries of the global north in only offering temporary statuses, like the one DACA provides, is precisely that immigrants will never be allowed to become full and included members of society (Motomura, 2006; Smith, 2022). In addition, these programs—and the individuals they protect—often face urgent legal threats as is the case with DACA. In this way, immigrant lives and their opportunities are forced into extreme precarity, and immigrant communities must endure different types of legal and physical violence.

Despite DACA's overwhelming success at incorporating into society those who did benefit from the program through things such as improved employment opportunities, this study's findings demonstrate that DACA's excluding nature acted as an internal border further preventing incorporation into U.S. society. The key findings of this article are testimony to how restrictive immigration policy can proliferate internal borders, which can be equally as harmful as more traditional forms of bordering. Beyond the exclusionary mechanisms embedded in immigration programs like DACA, internal borders are often created and maintained through inaction. For example, everyday border guards like the ones Carlos encountered at the police station would not issue necessary paperwork required in order to apply to the DACA program.

## Data availability statement

The datasets presented in this article are not readily available because this research data base is not allowed to be shared by IRB protocol. It is stored in the MAXQDA qualitative data analysis program. Requests to access the datasets should be directed to LS, lindaes@uci.edu.

## Ethics statement

The studies involving human participants were reviewed and approved by the Institutional Review Board (IRB) at the University of California, Irvine. The IRB waived the requirement for written informed consent to be obtained. Oral consent for participation was obtained and documented via digital recording.

## Author contributions

The author confirms being the sole contributor of this work and has approved it for publication.
